# Editorial: Novel Immunotherapies Against Envenomings by Snakes and Other Venomous Animals

**DOI:** 10.3389/fimmu.2020.01004

**Published:** 2020-05-22

**Authors:** Andreas Hougaard Laustsen, Stuart Ainsworth, Bruno Lomonte, R. Manjunatha Kini, Carlos Chávez-Olórtegui

**Affiliations:** ^1^Department of Biotechnology and Biomedicine, Technical University of Denmark, Lyngby, Denmark; ^2^Centre for Snakebite Research & Interventions, Liverpool School of Tropical Medicine, Liverpool, United Kingdom; ^3^Facultad de Microbiología, Instituto Clodomiro Picado, Universidad de Costa Rica, San José, Costa Rica; ^4^Department of Biological Sciences, Faculty of Science, National University of Singapore, Singapore, Singapore; ^5^Department of Pharmacology, Yong Loo Lin School of Medicine, National University of Singapore, Singapore, Singapore; ^6^Departamento de Bioquímica, Instituto de Ciências Biológicas, Universidade Federal de Minas Gerais, Belo Horizonte, Brazil

**Keywords:** venom, envenoming, antivenom, recombinant antivenom, toxin-neutralization, immunotherapy, antibody discovery, serotherapy

Globally, several million people fall victim to animal envenomings each year. These envenomings are caused by snakes, scorpions, spiders, bees, and other venomous animals (Pucca et al.), which are able to inflict bites and stings, which inject venoms, mixtures of protein-based toxins, into their victims and prey. Since the late eighteenth century, the first-choice treatment for severe animal envenomings has been antivenoms, which consist of polyclonal antibodies or antibody fragments isolated from the serum or plasma of animals hyper-immunized with venoms. These essential medicines are invaluable in saving the lives and limbs of patients worldwide. However, these “conventional” antivenoms are burdened with a number of disadvantages, such as a high content of non-neutralizing antibodies, a propensity to induce adverse reactions (including serum sickness and anaphylaxis), and a relatively high cost of manufacture compared to many other routinely used therapeutics (1). In recent decades, many new biotechnologies have emerged, which have been successfully employed to develop breakthrough therapies within oncology, autoimmunity, and infectious diseases. These biotechnologies present an opportunity for the field of envenoming therapy, and antivenom researchers have therefore started to apply these new approaches with the goal of increasing the efficacy of plasma-derived antivenoms through innovative immunization approaches (2), or by utilizing recombinant DNA technology and antibody discovery methodologies to generate recombinant antivenoms with improved efficacy and safety profiles.

This Research Topic presents two reviews and two original research articles that exhibit prominent and recent examples of the developments within novel immunotherapies against envenomings by snakes and other venomous animals. In Pucca et al., the history of envenoming therapies is reviewed with a focus on the key developments in the life sciences that led to the generation of the first antivenoms, as well as their subsequent refinement and optimization. It underscores the impact of basic immunological research within the field, as well as the contributions generated within antivenom research, which led to the development of immunological methods and novel therapeutics based on monoclonal antibodies that are now utilized across almost all fields of medicine. This review further provides perspectives on the advances in biotechnology in the last decades, which have influenced the academic research agenda in antivenom research, and discusses how different scientific breakthroughs in synthetic biology, antibody technologies, immunology, and molecular biology, such as phage display technology, may be exploited in the development of improved, next-generation envenoming therapies.

In de Castro et al., an example is provided of how knowledge of venom biology and new immunization approaches can be harnessed in the development of improved serotherapies against envenomings by *Micrurus* spp. (coral snakes). Here, a combined immunization protocol, using priming doses of *M. frontalis* venom and booster doses of synthetic B-cell epitopes derived from *M. corallinus* three-finger toxins and phospholipases A_2_, elicited a humoral response against both venoms in a rabbit model. The raised sera displayed relevant cross-reactivity against venoms from other *Micrurus* species (*M. altirostris, M. lemniscatus, M. spixii*, and *M. surinamensis)* and were demonstrated to be able to neutralize PLA_2_ activity of *M. frontalis* and *M. corallinus* venoms. *In vivo*, the sera completely protected mice from a challenge with 1.5 median lethal dose (LD_50_) of *M. corallinus* venom and protected 50% of mice challenged with 1.5 LD_50_ of *M. frontalis* venom.

Bailon-Calderon et al. describe a promising example of the utilization of antibody technologies. In their work, a set of nanobodies was developed from a library of a *Lama glama* immunized with the venom of *Bothrops atrox*, a viperid of utmost medical relevance in South America. Several of the obtained recombinant nanobodies showed neutralizing ability toward hemorrhagic and myotoxic venom components, which are responsible for major local tissue damage. The study further highlights the valuable potential of these innovative biotherapeutics, bringing them one step closer to clinical application.

Finally, Pucca et al. provide a comprehensive overview of the scientific literature surrounding bee venom toxinology and the treatment against bee stings. Here, the epidemiology of *Apis* spp. (bees) is presented with a special focus on the species *Apis mellifera* (Africanized bee), which is a hybrid species of the African and European honey bees that cause a significant number of severe envenomings, some of which have resulted in human fatalities. Further, all proteinaceous components of bee venom are presented with a discussion on the two main toxic components, phospholipase A_2_ and melittin, and their synergistic toxic function in victims. The review concludes with a detailing of how bee stings are currently only treated symptomatically, and that opportunities exist for developing recombinant antivenoms against bee envenomings.

The field of next-generation antivenoms has recently gained increased interest, and technological advancements now enable researchers to undertake the ambitious endeavor of developing improved therapeutic interventions against animal envenomings. This trend will likely continue for the years to come, as such efforts are officially supported by the recently published strategy from the World Health Organization (3). Next-generation antivenom research is still a rather nascent field. However, within the next two decades, it is predicted that this area of research will experience significant development and innovation.

## Author Contributions

AL prepared the first draft. All authors corrected the manuscript and provided revisions.

## Conflict of Interest

The authors declare that the research was conducted in the absence of any commercial or financial relationships that could be construed as a potential conflict of interest.
